# Unlike in Drosophila Meroistic Ovaries, Hippo Represses Notch in *Blattella germanica* Panoistic Ovaries, Triggering the Mitosis-Endocycle Switch in the Follicular Cells

**DOI:** 10.1371/journal.pone.0113850

**Published:** 2014-11-26

**Authors:** Paula Irles, Maria-Dolors Piulachs

**Affiliations:** Institut de Biologia Evolutiva (CSIC - Universitat Pompeu Fabra), Barcelona, Spain; Instituto Gulbenkian de CiÃancia, Portugal

## Abstract

During insect oogenesis, the follicular epithelium undergoes both cell proliferation and apoptosis, thus modulating ovarian follicle growth. The Hippo pathway is key in these processes, and has been thoroughly studied in the meroistic ovaries of *Drosophila melanogaster*. However, nothing is known about the role of the Hippo pathway in primitive panoistic ovaries. This work examines the mRNA expression levels of the main components of the Hippo pathway in the panoistic ovary of the basal insect species *Blattella germanica*, and demonstrates the function of Hippo through RNAi. In Hippo-depleted specimens, the follicular cells of the basal ovarian follicles proliferate without arresting cytokinesis; the epithelium therefore becomes bilayered, impairing ovarian follicle growth. This phenotype is accompanied by long stalks between the ovarian follicles. In *D. melanogaster* loss of function of Notch determines that the stalk is not developed. With this in mind, we tested whether Hippo and Notch pathways are related in *B. germanica*. In Notch (only)-depleted females, no stalks were formed between the ovarian follicles. Simultaneous depletion of Hippo and Notch rescued partially the stalk to wild-type. Unlike in the meroistic ovaries of *D. melanogaster*, in panoistic ovaries the Hippo pathway appears to regulate follicular cell proliferation by acting as a repressor of Notch, triggering the switch from mitosis to the endocycle in the follicular cells. The phylogenetically basal position of *B. germanica* suggests that this might be the ancestral function of Hippo in insect ovaries.

## Introduction

The difference between the rate of cell proliferation and cell death is a determinant of tissue growth in all organisms, and a precise control of these processes is essential to reach a correct development. The Hippo pathway, a kinase cascade, is a key regulator of tissue growth in animals ranging from premetazoans to humans [1]–[4]. A misregulation of this pathway leads to organ overgrowth that may culminate in tumorigenesis [5], [6].

The control that Hippo pathway exerts on epithelial cell proliferation has been thoroughly studied in the meroistic ovaries of *Drosophila melanogaster* [7], [8]. In this species, *hpo* mutants show an overproliferation of the posterior follicular cells giving rise to a bilayered, and sometimes multilayered, epithelium [7]–[9]. All these studies have highlighted multiple essential functions of the Hippo pathway in insect oogenesis, some of them related with the oocyte axis specification. Thus, in *hpo* or *wts* mutants, *bicoid, grk and osk* were mislocalized due to changes in the microtubule organization affecting the anterior-posterior axis determination in the oocyte [7]. Also, it has been described an important interaction between Hippo and Notch pathways, as Notch is crucial in the follicular cells to coordinate the switch from mitosis to endocycle [10]. The Notch pathway is attenuated when Hpo signaling is disrupted, which affects oocyte polarity and follicular cell differentiation [8], [9]. Then, the loss-of-function of Notch determines that the stalk and polar cells cannot develop [8], [9], [11]. The role of Notch pathway has also been studied in the meroistic telotrophic ovary of *Tribolium castaneum* [12], where in contrast to *D. melanogaster*, Notch participates in maintaining the cells in an undifferentiated state.

**Figure 1:**
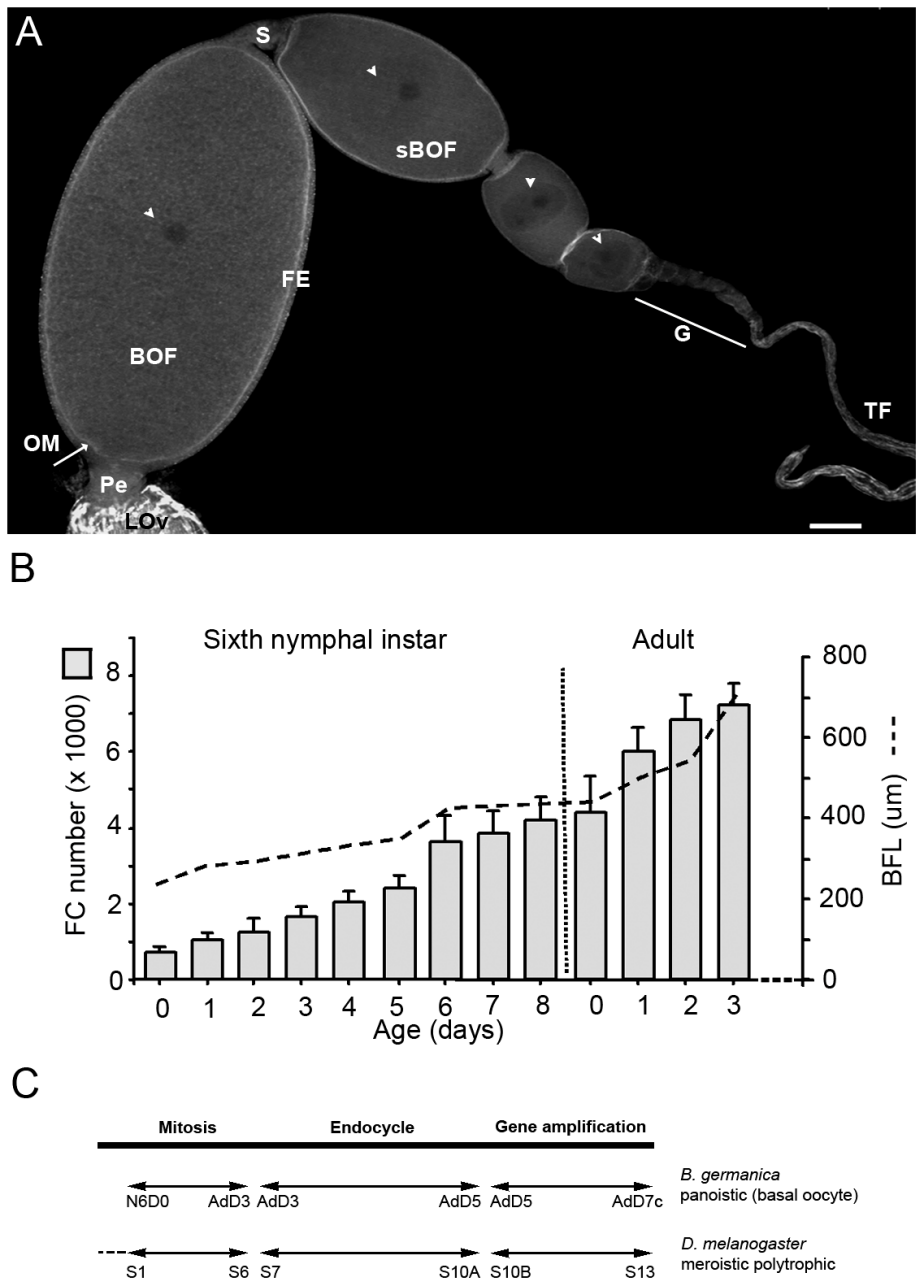
Panoistic ovariole of *B. germanica* and dynamics of follicular cell proliferation. (A) Panoistic ovariole from a 0-day old *B. germanica* adult female. Only the basal ovarian follicle (BOF) grows and matures in any one gonadotrophic cycle. Germinal vesicles are indicated by the arrowheads. Pe: pedicel, OM: oocyte membrane, FE: follicular epithelium; sBOF: sub-basal ovarian follicle, G: germaria, TF: terminal filament. LOv: lateral oviduct, s: stalk. Scale bar: 50 µm. (B) Follicular cell number and length of the basal ovarian follicle (BFL) during the sixth nymphal instar and previtellogenic period in the adult. Data are expressed as mean ± s.e.m. C: Progression of follicular cell cycle in the basal ovarian follicle from the first day of the sixth instar nymph (N6D0) to chorion formation in the adult (Ad7c), compared to the progress of the follicular cell cycle through the different eggs chambers (stages, S) in one ovariole of *D. melanogaster*.

The role of the Hippo pathway in other insect ovary types, however, remains a mystery. This paper unveils the role of the Hippo pathway in the primitive panoistic ovary of the cockroach *Blattella germanica* (L), a phylogenetically basal hemimetabolous species in which reproduction is regulated by juvenile hormone. In panoistic ovaries, the ovarian follicle is characterized by the absence of nurse cells, and consists of an oocyte surrounded by a follicular epithelium [13] (Figure 1A). The latter plays an important function in the growth of the developing oocyte [14]–[16] and in determining the final size and shape of the ovarian follicle [17]. In *B. germanica* oogenesis, the basal oocyte is the only one to mature in the gonadotrophic cycle; the other ovarian follicles remain in the vitellarium to take up the basal position in following cycles. It is in the sixth (last) nymphal instar when the basal ovarian follicle becomes active and starts to grow. At the beginning of the instar, follicular cells actively proliferate, increasing from 1000 just after moulting to 4500 at the beginning of the adult stage (Figure 1B). In 3-day-old adult females, the follicular epithelium reaches the maximum number of cells (around 7500) and the mitosis-endocycle switch is activated. The female thus enters the vitellogenic period and the basal oocyte begins to take up protein [18], [19], resulting in the exponential growth of the basal ovarian follicle. After vitellogenesis, the follicular cells secrete the different chorion components [15].

The regulation of follicular cell proliferation is crucial in understanding the dynamics of follicle growth in panoistic ovaries, which involves the Hippo pathway [8], [9], [11]. Unlike that described in *D. melanogaster*, a highly modified insect species, the present work shows that, in *B. germanica*, Notch activity increases when Hippo mRNA leves are depleted. This prevents the switch from mitosis to the endocycle in follicular cells and gives rise to long-stalk phenotypes. Given the phyllogenetically basal nature of *B. germanica*, the repressive action of Hippo upon Notch might be the ancestral mechanism regulating follicle cell proliferation in insect ovaries.

## Material and Methods

### Cockroach colony and animal sampling

Adult females of the cockroach *B. germanica* (L.) were obtained from a colony fed *ad libitum* on Panlab dog chow and water, and reared in the dark at 29±1°C and 60–70% relative humidity. Freshly ecdysed adult females were selected and used at appropriate ages. Mated females were used in all experiments (the presence of spermatozoa in the spermatheca was assessed to confirm that mating had occurred). All dissections and tissue samplings were performed on carbon dioxide-anaesthetized specimens under Ringer's saline (1.8 mM CaCl2, 154 mM NaCl, 2.68 mM KCl and 2.38 mM NaHCO3).

### Cloning of Hippo cDNA

An 1884 bp fragment of *B. germanica* Hippo (BgHpo) was isolated from mRNA libraries representatives of different organs and stages of cockroach development (previously obtained in our laboratory). The fragment contained the coding region from the amino acid at position 30 through to the poly (A) tail, but lacked the N-terminal end. To complete it, 5′ rapid amplification of cDNA ends (RACE) was performed on RNA extracted from adult ovaries using the FirstChoice RLM-RACE Kit (Ambion, Huntingdon, Cambridgeshire, UK) according to the manufacturer's instructions. The sequence was amplified, cloned into the pSTBlue-1 vector (Novagen, Madison, WI, USA) and sequenced. Table S1 shows the primer sequences used in this amplification.

### RNA extraction and expression studies

Total RNA was isolated using the GenElute Mammalian Total RNA Kit (Sigma, Madrid, SPAIN). A total of 400 ng from each RNA extraction was treated with DNAse (Promega, Madison, WI, USA) and reverse transcribed with Superscript II reverse transcriptase (Invitrogen, Carlsbad CA, USA) and random hexamers (Promega). RNA quantity and quality were estimated by spectrophotometric absorption at 260/280 nm in a Nanodrop Spectrophotometer ND-1000 (NanoDrop Technologies, Wilmington, DE, USA). The expression patterns of Kibra (BgKibra), Merlin (BgMer), expanded (BgExp), Hippo (BgHpo), salvador (BgSav), warts (BgWts), MATS (BgMats), yorkie (BgYki) and scalloped (BgSd) were determined by quantitative real time PCR (qRT-PCR) in the sixth instar nymph stage and during the first gonadotrophic cycle in the adult stage. Pools of 2 to 6 ovary pairs for each chosen adult age were used. The primers used in qRT-PCR expression studies were designed using Primer3 v.0.4.0 software [20] (sequences in Table S1). The actin-5c gene of *B. germanica* (Accession number AJ862721) was used as a reference for expression studies, and the eukaryotic initiation factor 4A, BgEIF4a (Accession number HF969254), for functional studies. PCR reactions were performed using the IQTM SYBR Green Supermix Kit (BioRad, Barcelona, SPAIN) with 200 nM of each specific primer (performed in triplicate). Amplification reactions were performed as follows: 95°C for 2 min, 40 cycles of 95°C for 15 s, and 60°C for 30 s, using the MyIQ Single Color RTPCR Detection System (BioRad). After the amplification phase, a dissociation curve was produced to ensure that only one product was formed [15].

### RNAi experiments

To knockdown BgHpo and assess the specificity of the phenotype, two dsRNAs (dsBgHpo-1 and dsBgHpo-2) were designed. The first, dsBgHpo-1, was 406 bp in length and comprised the subdomains V-IX of the protein serine/threonine kinase catalytic domain (nucleotides 382-788). The second, dsBgHippo-2, with 420 bp (nucleotides 1294-1714), belongs to a region where no conserved domain has been detected. Both RNAis were independently injected (1 µg/µl) into newly-emerged sixth nymphal instar females (N6D0). Since the same ovary phenotype was found using both dsBgHpo-1 and dsBgHpo-2, all RNAi treatments are hereafter referred to as dsBgHpo. To deplete BgWts, a 255 bp dsRNA (dsBgWts) was designed overlapping part of the catalytic domain from the Serine/Threonine Kinases, Large Tumor Suppressor subfamily proteins. dsBgWts (1 µg/µl) was injected into 0-day-old sixth nymphal instar females. For experiments on BgN knockdowns, a 363 bp dsRNA (dsBgN) was designed that encompassed a fragment of the Ankyrin domain of the protein. dsBgN (1 µg/µl) was injected into 6-day-old sixth nymphal instar females. To reduce the levels of BgN in dsBgHpo-treated females, double sequential RNAi treatment was performed on sixth nymphal instar females. First, dsBgHpo was injected into 0-day-old nymphs, and dsBgN injected when they reached 6 days. A 92 bp non-coding sequence from the pSTBlue-1 vector (dsMock) was used as control dsRNA (same dose and conditions as in dsRNA treatments). All four cDNAs were amplified by PCR and cloned into the pSTBlue-1 vector. Single stranded sense and antisense RNAs were obtained by transcription *in vitro* using either SP6 or T7 RNA polymerases from the respective plasmids, and resuspended in water. To generate the dsRNAs, equimolar amounts of sense and antisense RNAs were mixed, heated at 95°C for 10 min, cooled slowly to room temperature, and stored at −20 °C until use. The formation of dsRNA was confirmed by running 1 µL of the reaction products on 1% agarose gels. dsRNAs were suspended in diethyl pyrocarbonate-treated water and diluted in Ringer's saline.

### Immunohistochemistry

Removed ovaries were immediately fixed in paraformaldehyde (4% in PBS) for 2 h, washed in PBT (PBS; 0.3% Triton-X100), treated with 50 µg/ml proteinase K for 2 min, washed for 2 min in 2 mg/ml glycine in PBT, washed for 10 min in PBT, and fixed again for 20 min in the same solution. After three washes with PBT, the tissues were placed in PBTBN (PBS with 0.1% Triton X-100, 0.5% BSA and 5% normal goat serum) for 1 h at room temperature. They were then incubated overnight at 4°C with the primary antibodies rabbit anti-PH3 (Cell Signaling Technology, Danver, MA, USA) (1∶250), mouse anti-ß-tubulin (1∶50), and mouse anti-NICD (Developmental Studies Hybridoma Bank, University of Iowa, Department of Biology, Iowa City, IA, USA) (1∶100). The tissues were washed with PBTBN three times and incubated for 2 h with either Alexa-Fluor 647 conjugated donkey anti-rabbit IgG or Alexa-Fluor 488 conjugated goat anti-mouse IgG secondary antibody (Molecular Probes, Carlsbad, CA, USA), both diluted to 1∶400 in PBTBN. In addition, ovaries were incubated at room temperature for 20 min in 300 ng/ml phalloidin-TRITC (Sigma) and then for 5 min in 1 µg/ml DAPI (Sigma) PBT. After three washes with PBT, ovaries were mounted in Mowiol (Calbiochem, Madison, WI, USA) and observed using a Zeiss AxioImager Z1 microscope (Apotome) (Carl Zeiss MicroImaging).

The number of cells in the follicular epithelia was estimated applying the function described in Pascual et al. [21].

### Statistics

The data are expressed as means ± standard error of the mean (s.e.m.). Comparisons between tissue samples were made using the non-parametric Mann-Whitney test, employing GraphPad Prism 6 software. Comparisons between treatment and control groups were made using the Pair-Wise Fixed Reallocation Randomization Test (which makes no assumptions about distributions) [22], employing REST 2008 v. 2.0.7 software (Corbett Research).

## Results

### The complete set of Hippo pathway components is expressed in the B. germanica ovary

**Figure 2:**
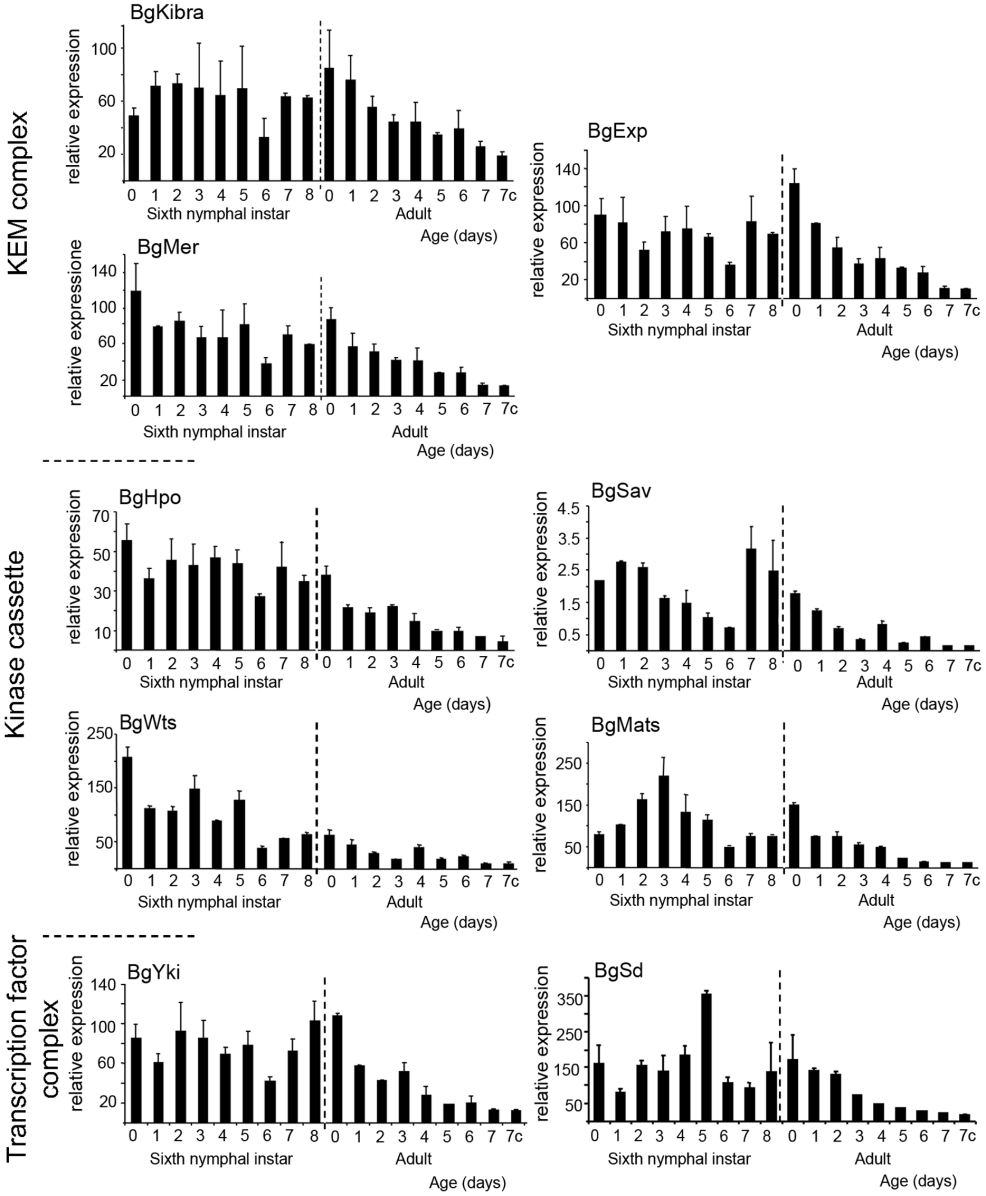
Expression pattern of the Hippo pathway components in ovaries of *B. germanica*. mRNA expression pattern of the components of the Hippo pathway in ovaries in sixth nymphal instar and adult females during the first gonadotrophic cycle. The dashed line indicates the moult to adult. Data represent copies of mRNA per 1000 copies of BgActin-5c (relative expression) and are expressed as the mean ± s.e.m. (n = 3).

The mRNA expression pattern of the Hippo pathway components (Kibra [BgKibra], Merlin [BgMer] and expanded [BgExp] [part of the apical complex], the kinase cassette composed of hippo [BgHpo], salvador [BgSav], warts [BgWts], MATS [BgMats], and the transcriptional co-activator yorkie [BgYki] and its partner scalloped [BgSd]), was examined in the *B. germanica* ovary in sixth (last) nymphal instar and young adult females (Figure 2). In the last nymphal instar the basal ovarian follicles complete their maturation, and follicular cells show the highest proliferation rate, which coincides with the highest rate of expression of all the components of the Hippo pathway. Of note, BgKibra, BgExp, BgSav and BgYki show especially high expression during the days closest to the imaginal moult. The scaffold protein BgMats and the coactivator BgSd show well-defined peaks on days 3 and 5 of the sixth nymphal instar, respectively (Figure 2). After the moult to adult, when basal follicles enter in vitellogenesis and become ready to uptake proteins, the expression of all the components of the Hippo pathway steadily decrease as the basal ovarian follicles mature. The lowest expression levels are reached just before oviposition, which take place 7 days after the emergence of the adult. These patterns clearly indicate that the main changes in the expression of Hippo pathway components occur during the sixth instar rather than during the adult stage, when the expression of all these genes gradually decreases.

### Hippo is required for basal ovarian follicle growth and the mitosis-endocycle switch in follicular cells

**Figure 3:**
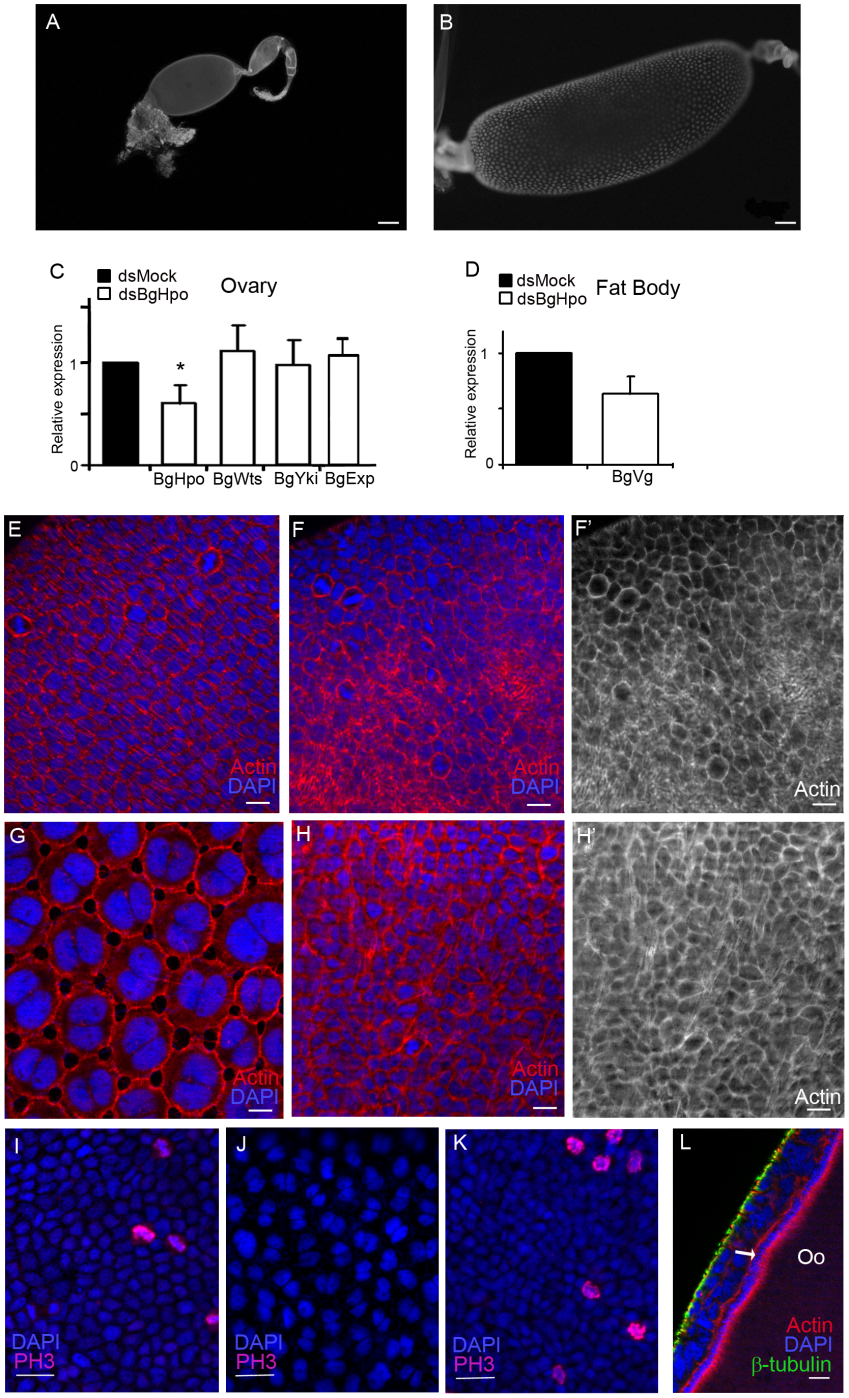
BgHpo controls follicular cell proliferation in developing follicles. Ovarioles from 5-day-old adult females treated with dsBgHpo (A), or dsMock (B). The posterior end of the ovarian follicles is towards the left. Scale bar: 100 µm. (C) Expression of BgHpo, BgWts and BgYki in ovaries from 5-day-old adult dsMock and dsBgHpo-treated females. BgHpo is significantly depleted in treated ovaries [P(H1)  = 0.004]. (D) Expression of BgVg in fat body from 5-day-old adult dsMock and dsBgHpo-treated females (no significant difference [P(H1)  = 0.098]. Data represent normalized values against the control (reference value  = 1) (n = 3). (E, F, F′) Follicular epithelium from 0-day-old adult females in which cells are dividing actively in dsMock- (E and Figure S1) and dsBgHpo- (F, F′) treated females. Scale bar: 10 µm. (G, H, H′) Follicular cells from 5-day-old adult females. In the dsMock (G and Figure S1) females, mitosis is arrested and patency is already apparent. In the dsBgHpo (H, H′) females, the cells are still dividing and the cytoskeleton is disorganized Scale bar: 10 µm. (I, J, K) Follicular cells in adult ovarioles labelled with an anti-phospho-histone 3 antibody (PH3). (I) Follicular cells from 0-day-old and (J) 5-day-old dsMock-treated females. (K) Follicular cells from a 5-day-old dsBgHpo-treated female. Scale bar: 20 µm. (L) Optical section of (K), the arrow indicates the layer of endosymbiont bacteriocytes. Oo: ooplasm. Scale bar: 10 µm.

To investigate whether *hpo* regulates the transition from mitosis to the endocycle in the panoistic ovaries of *B. germanica*, BgHpo transcripts were depleted by RNAi via the injection of dsBgHpo into 0-day-old sixth instar nymphs. In 5-day-old adults, the basal ovarian follicles in dsBgHpo-treated females (0.44±0.04 mm length; n = 27, Figure 3A) remained as small as in 0-day-old adult dsMock-treated females, and never reached the length of those observed in 5-day-old adult dsMock-treated females (1.55±0.08 mm; n = 12; P<0.0001; Figure 3B). Despite the strong size reduction of the basal ovarian follicles, the mRNA levels of BgHpo were significantly downregulated a 50% (fold change 1.67; Figure 3C) in the ovaries of 5-day-old adult females treated with dsBgHpo. Moreover, the depletion of BgHpo mRNA had no effect on the expression of BgYki, BgWts (downstream components of the pathway) or BgExp (an upstream component) (Figure 3C).

The follicular cells of 0-day-old adult dsBgHpo-treated females resembled those of dsMock-treated females (cuboid and tightly packed Figure 3 F, and Figure 3E and S1, respectively) and showed a similar number of cells undergoing mitosis. However, the arrangement of the F-actin microfilaments of the cytoskeleton was irregular, with random accumulations in different areas of the epithelium (Figure 3, F and F′). In 5-day-old dsMock-treated adult females, the follicular cells were larger, binucleated and the actin microfilaments were associated with cell membranes displaying lateral extensions, and therefore allowing large intercellular spaces (patency) (Figure 3G, and Figure S1). In contrast, in BgHpo knockdown females the cells remained smaller, showed a single nucleus, and maintained close contact each other (Figure 3H). Of note, in the cytoplasm of follicular cells of BgHpo knockdowns, the actin filaments became disorganized and showed strong labelling (Figure 3H′).

**Figure 4:**
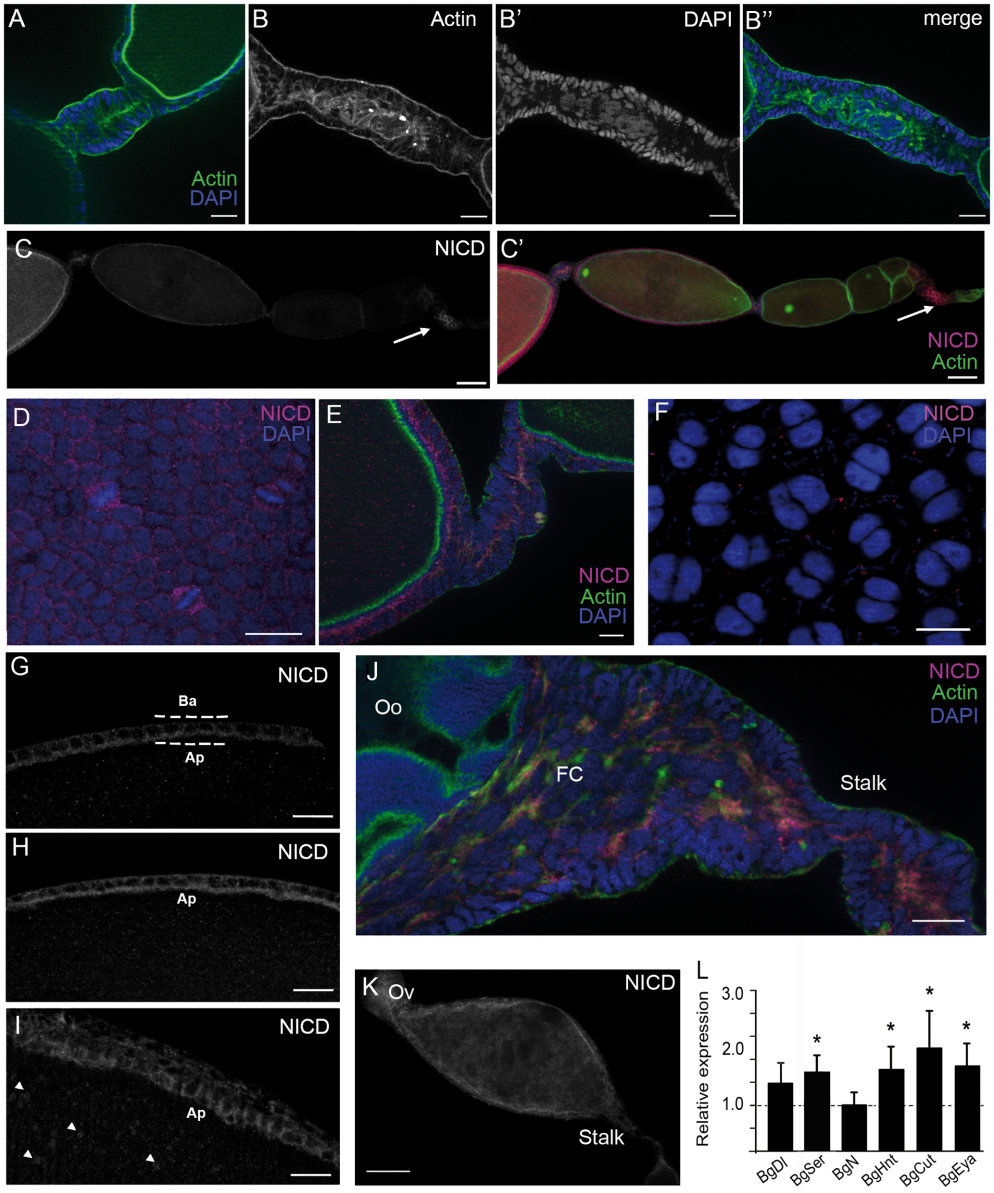
Loss of BgHpo generates long-stalks activating BgN. (A) Stalk from a 5-day-old dsMock adult female. F-actin labelling in the apical tips of the cells. Scale bar: 20 µm. (B, B′, B″) Long-stalk phenotype from a 5-day-old dsBgHpo adult female. (B) F-actin labelling is concentrated in the central axis of the stalk. (B′) The cells are smaller but greater in number. (B″) Merging of (B) and (B′). Scale bar: 20 µm. (C) Ovariole from 0-day-old adult female showing NICD localization. (C′) Merge image of NICD and F-Actin labelling. The arrow indicates the labelling in the germarium. Scale bar: 50 µm. (D) Follicular cells from 0-day-old dsMock adult female; the dividing cells show strong NICD labelling. Scale bar: 20 µm. (E) Stalk from 0-day-old dsMock adult female showing NICD labelling. Scale bar: 10 µm. (F) follicular cells from 5-day-old dsMock adult female. Scale bar: 20 µm (the optical section of 5-day-old dsMock adult female is showed in Figure 5D). (G, H, I) Optical section of a basal ovarian follicle showing the different localization of NICD between the apical (Ap) and basal pole (Ba) of the follicular cells. (G) 0-day-old dsMock adult female. (H) 0-day-old and (I) 5-day-old dsBgHpo adult female. Arrowheads in (I) indicate NICD accumulation in the ooplasm. Scale bar: 20 µm. (J) Stalk in a 5-day-old dsBgHpo adult female ovariole. Oo: ooplasm, FC: follicular cells. Scale bar: 10 µm. (K) Basal ovarian follicle from a 5-day-old dsBgHpo adult female. Ov: oviduct. Scale bar: 50 µm. (L) mRNA expression of different components of the Notch pathway in ovaries of 5-day-old dsBgHpo adult females. BgSer, BgHnt, BgCut and BgEya were upregulated (P(H1)  = 0.0001, 0.010, 0.005 and 0.001 respectively); BgDl and BgN were not significantly affected. Data represent normalized values against dsMock controls (reference value  = 1, dashed line) (n = 3). In all images the posterior pole of the basal follicle is towards the left.

Further, while mitosis was arrested and the follicular cells became binucleated and entered the endocycle in 5-day-old dsMock-treated adults (Figure 3J), in 5-day-old BgHpo knockdown females these cells kept dividing (Figure 3K), with 30% more cells undergoing mitosis than in 0-day-old dsMock-treated females (Figure 3I). As a consequence of this overproliferation, the follicular epithelium became bilayered (Figure 3L, 4I and S2), mainly in the lateral and posterior regions of the basal ovarian follicle. This impedes the oocyte to reach its normal size and provokes the membrane folding (Figure S2). We attempted to quantify the cell number in the follicular epithelium of dsBgHpo-treated females, and despite the small size of the ovarian follicle, we found that in the most external layer, the number of cells was similar to that present in a dsMock-treated female. However, considering that the follicular epithelium in these treated females was bilayered (Figure 3L, 4I and S2), the total number of cells in dsBgHpo basal follicles would be approximately the double than in dsMock females. This overproliferation in dsBgHpo-treated females impaired the growth of the basal ovarian follicle, as it prevented vitellogenin (BgVg) uptake. Interestingly, BgVg was synthesized in the fat body of dsBgHpo-treated females, although at a somewhat lower levels compared to dsMock-treated females (Figure 3D).

### BgHpo depletion produces long stalks

A longitudinal optical section of *B. germanica* stalk (Figure 4A) shows a tubular structure formed by a monolayer of differentiated follicular cells, with the actin microfilaments distributed mainly in the apical tips of the stalk cells and extending towards the lateral surface. Strikingly, the stalks in all dsBgHpo-treated females were unusually long, between 181% and 205% longer than in dsMock, a defect observed primarily between the basal and sub-basal follicles (Figure 4B). Although the stalks in BgHpo knockdowns were longer and cell numbers greater than in the dsMock group, no mitosis was detected (Figure 4B′). The cells were smaller and not well arranged into a monolayer, and the actin filaments were randomly distributed (Figure 4, B and B″).

This unexpected phenotype led us to confirm it by depleting another component of the pathway. We depleted BgWts as a part of the kinase cassette, downstream of Hippo. In 5-day-old dsBgWts-treated females the basal ovarian follicles were small and showed long stalk (Figure S3 C-E). The follicular cells were small and appeared very close to each other, without intercellular spaces, and they continued dividing as mitosis were detected with anti-PH3 labeling (Figure S3 F).

**Figure 5:**
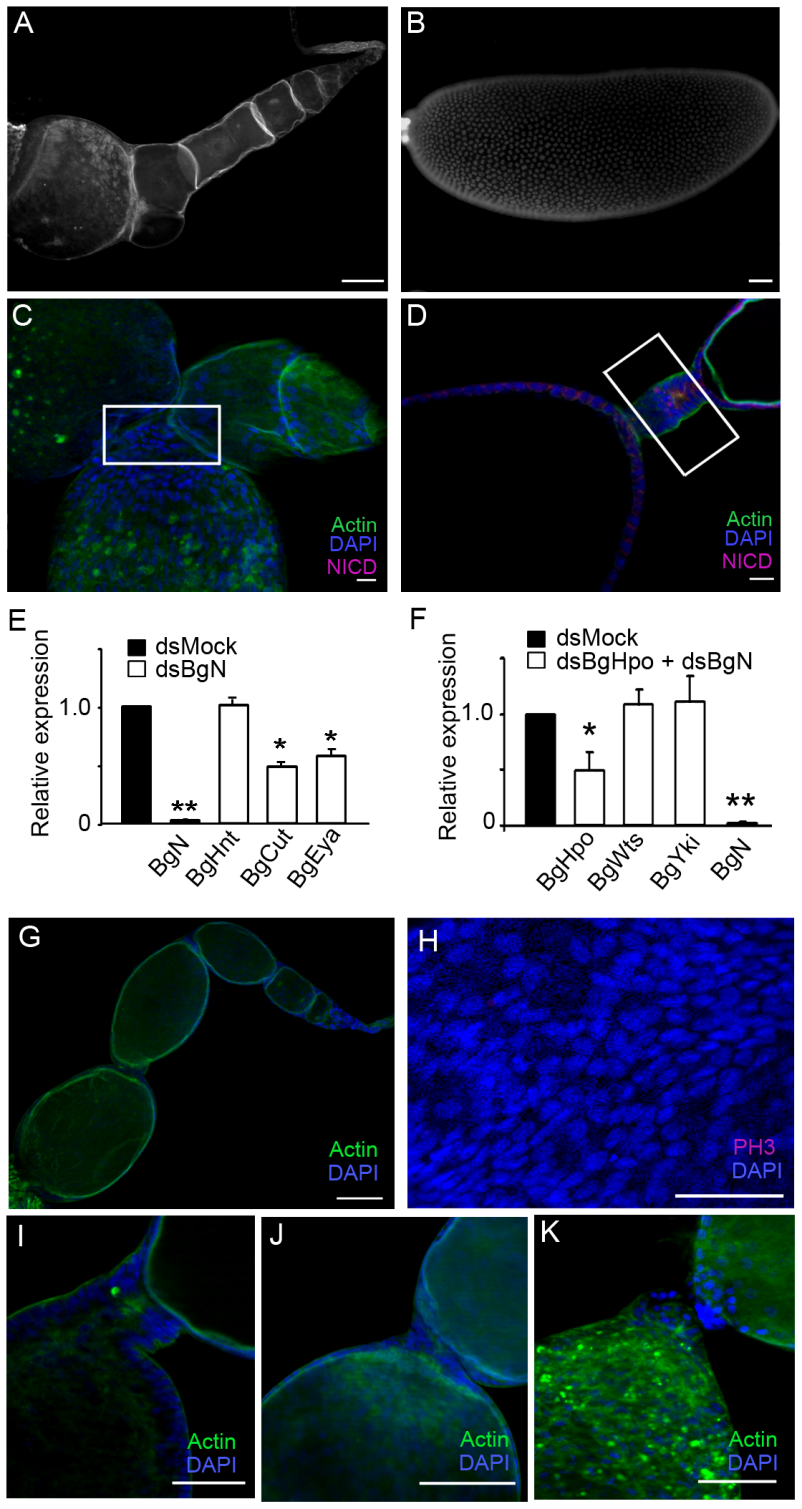
Notch depletion suppresses the stalk and reduces the long stalk phenotype in dsBgHpo knockdowns. Ovariole from a 5-day-old adult female treated with dsBgN (A), and a basal ovarian follicle from a dsMock 5-day-old adult female (B). Scale bar: 100 µm. (C, D) Localization of Notch in ovarioles of 5-day-old adult dsBgN (C) and dsMock (D) females showing the position of the stalk (white box). In (C) there is no stalk. Notch was detected with an anti-NICD antibody. Scale bar: 20 µm. (E) mRNA relative expression of BgN, BgHnt, BgCut and BgEya in ovaries from 5-day-old adult dsMock- and dsBgN-treated females. BgN, BgCut and BgEya were significantly down-regulated (P(H1)  = 0.0001) in dsBgN-treated ovaries. (F) mRNA relative expression of BgHpo, BgWts, BgYki and BgN in ovaries from 5-day-old adult dsMock and dsBgHpo + dsBgN-treated females. BgHpo and BgN were significantly reduced (P(H1)  = 0.047 and 0.028 respectively). Data represent normalized values against the control (reference value  = 1) (n = 3). (G) Ovariole from a 5-day-old, adult, double knockdown female (dsBgHpo + dsBgN). Scale bar: 100 µm. (H) Follicular epithelium where no mitoses were detected in opposite to dsBgHpo treatment (see Figure 3K). Scale bar: 50 µm. (I–J) Different range of stalk phenotypes resulting from the double knockdown. Scale bar in I and K: 50 µm, in J: 100 µm. In all images the posterior end of the basal ovarian follicle is towards the left, except in (C) in which it is towards the bottom.

A long-stalk phenotype has been described in *D. melanogaster* when active Notch and Delta are expressed constitutively in ovaries [23], [24]. This suggests that, in *B. germanica*, Notch would be affected by the depletion of BgHpo. The involvement of Notch in the long-stalk phenotype in dsBgHpo females was examined using an antibody against the intracellular domain of *D. melanogaster* Notch (NICD). In ovarioles of 0-day-old dsMock-treated specimens, Notch labelling was detected in the basal ovarian follicle, in the stalk between the basal and sub-basal ovarian follicles, and in the germarium (Figure 4, C and C′). In the basal ovarian follicle, NICD was located differently in the apical pole of the follicular cells (Figure 4, E and G). However, labelling was more conspicuous in cells that were dividing, with NICD distributed throughout the cytoplasm (Figure 4D). In the stalk, of 0-day-old dsMock-treated females Notch was also mainly located in the apical pole of the cells (Figure 4E). However, in follicular cells from 5-day-old dsMock ovarian follicles NICD labelling was very faint (Figure 4F and 5D). NICD was also strongly apparent in the apical pole of the follicular cells of basal ovarian follicles from 0-day-old dsBgHpo adult females (Figure 4H). Moreover, in 5-day-old treated females, Notch continued to be present, and the labelling was extended to the lateral and the basal pole of the follicular cells (Figure 4I). An accumulation of NICD as discrete spots throughout the ooplasm, probably localized in vesicles, was also observed (Figure 4I). Unfortunately, the thickness of the full vitellogenic basal follicle in 5-day-old dsMock-treated specimens made the observation of Notch protein within the oocyte impossible. In 5-day-old dsBgHpo-treated females, NICD was abundant through the entire stalk (Figure 4J). A gradient of NICD labelling was in fact seen along the longitudinal axis of the basal ovarian follicle with more at the posterior pole (including the pedicel and the lateral oviduct), decreasing towards the anterior pole (Figure 4K).

Since Notch protein was detected in ovaries of 5-day-old adult BgHpo-treated females, the mRNA levels for a selection of Notch pathway components and Notch-dependent differentiation markers were measured. This selection, chosen with reference to the information available in *D. melanogaster*, includes Hindsight (BgHnt), Cut (BgCut) and Eye Absent (BgEya). No change was seen in Notch (BgN) mRNA levels, and no significant changes were observed in the expression of the ligand Delta (BgDl). However, the expression of Serrate (BgSer), the other ligand of Notch, was significantly upregulated (1.79 fold-change; Figure 4L). The increase in the expression of BgSer suggests a continuous activation of Notch signaling in these 5-day-old dsBgHpo-treated females. In addition, in these 5-day-old adult BgHpo-treated females BgHnt, BgCut, and BgEya were upregulated, with a 1.7 fold-change, 2.2 fold-change and 1.8 fold-change, respectively (Figure 4L).

### Notch determines the correct structure of the stalk

To assess the function of Notch in the development of the stalk in panoistic ovaries, BgN was depleted by RNAi. dsBgN was injected into 6-day-old sixth instar females and the basal ovarian follicle was observed in 5-day-old adults. In BgN knockdowns, the basal ovarian follicles were significantly smaller (0.28±0.01 mm, n = 16; Figure 5A) than in the dsMock group (1.52±0.07 mm, n = 10; P<0.0001; Figure 5B), and even smaller than in 0-day-old dsMock-treated adult females (0.47±0.04 mm, n = 12; P<0.0001). Moreover, while the basal ovarian follicles of dsMock females showed the typical elliptical shape (Figure 5, B and D), those of 5-day-old dsBgN-treated females exhibited a sub-spherical shape (Figure 5A) the ovarioles showed no stalks (Figure 5, A and C) and were no follicular cells in mitosis (not shown). In the ovaries of dsBgN-treated females, the mRNA levels of BgN, BgCut and BgEya were significantly reduced (42-, 2.1- and 1.72-fold respectively; Figure 5E), indicating that Notch activates Cut and Eya. Interestingly, BgHnt expression was not affected (Figure 5E), suggesting that Notch does not act directly on Hnt to regulate Cut. In dsBgN-treated females, Notch labelling was not observed in any ovariole (Figure 5C), while in the basal ovarian follicles of dsMock females, faint labelling was observed, mainly along the stalks (Figure 5D). These results show that Notch is required for normal stalk formation in the panoistic ovaries of *B. germanica*.

### Notch depletion recovers the phenotypes resulting from a reduction in Hpo

In the ovaries of BgHpo knockdowns, the follicular cells were unable to switch to the endocycle (Figure 3, H and L) and the stalks were longer than in ovarioles from the dsMock group (Figure 4B′) due to the continuous expression of BgN. To demonstrate a relationship between BgHpo and BgN, double sequential knockdowns of BgHpo and BgN were prepared. The corresponding mRNA levels were measured in 5-day-old adult females, which showed the expression of BgHpo to be significantly reduced (2-fold), and BgN to be dramatically depleted (38.5-fold; Figure 5F). These results were similar to those obtained for individual treatments (Figure 3C and 5E). Five-day-old double knockdowns females have ovarioles (Figure 5G) that showed reduced, and sometimes absent, stalks (Figures 5, I–J). In addition, 30% of the double knockdown females (n = 15) showed only a few dividing follicular cells, while the remaining 70% showed no mitosis (Figure 5H), a remarkable phenotype when compared with the continuous cell division found in dsBgHpo basal ovarian follicles (Figure 3L). These results suggest that low levels of BgN are necessary for the correct stalk formation and to stop the mitosis program in follicular cells.

## Discussion

In hemimetabolan insects, oogenesis proceeds gradually throughout post-embryonic development, and it is in the adult when the oocytes complete growth and reach maturity. One characteristic of *B. germanica* oogenesis is that only the basal oocyte develops in each gonadotrophic cycle [25]. This oocyte begins maturation in the last nymphal instar, expressing most of the genes that will be necessary for its development and the formation of the future embryo [25], [26]. During the last nymphal instar the follicular epithelium that surrounds the basal oocyte shows the highest rate of proliferation, coinciding with the highest expression of the main components of the Hippo pathway. Later, in the adult, when the basal oocyte is ready to uptake yolk proteins, proliferation is arrested in the follicular epithelium and mRNA levels of the Hippo pathway components gradually decrease, reaching their lowest levels just before oviposition. According to the mRNA expression profiles, it seems clear that the transcriptional regulation for each component of the pathway should be different. But actually, the activation or deactivation of the Hippo pathway is regulated post-translationally by phosphorylation [4], [27], [28].

The cell proliferation and organ size control function exerted by *hpo* in many organisms [1], [2], [29] is seen in the ovaries of *B. germanica*. The present results show that BgHpo controls the transition from mitosis to endocycling and, as a consequence, determines the total number of cells surrounding the basal ovarian follicle, and thus its final, optimal size. However, the lack of growth observed in the basal ovarian follicles of dsBgHpo-treated females contrasts with the typical overgrowth observed in other tissues (e.g., the eye and wing imaginal discs) in *D. melanogaster hpo*, *sav* and *wts* mutants [30], [31]. Despite this lack of growth, the follicular epithelium becomes bilayered in dsBgHpo-treated females, mainly in the lateral and posterior regions of the basal ovarian follicle, whereas in *D. melanogaster hpo* mutants [7], [9] although multiple-layers were observed, most are localized in the anterior and posterior regions of the egg chamber and not in the lateral part. The overproliferation in the follicular epithelium and the change in the size and form of the cells in dsBgHpo-treated females resulted also in a disorganization of the actins. They appeared spread through the cytoplasm and not associated with cell membranes as usually are in 5-day-old females to modulate the follicular cell shape, thus allowing patency to appear [17]. Actin dynamics is very important to maintain cell shape and integrity of the tissues by establishing links with the neighboring cells that can be translated to proliferation, differentiation or apoptosis [32]. A link between actin cytoskeleton and the Hippo pathway was described in *D. melanogaster*, as inhibition of actin polymerization results in Yki activation and a tissue overgrowth, a function evolutionary conserved as it is maintained in vertebrates [28], [33].

The cell overgrown in the follicular epithelium of dsBgHpo-treated females does not impede oocyte growth as they show their membrane folded, but the oocyte cannot uptake circulating BgVg; since it could not reach the oocyte membrane and cannot be taken up by its specific receptor [19].

The control of the mitosis-endocycle switch in follicular cells has been associated with the Notch pathway in *D. melanogaster* [34], [35] as Notch signaling is attenuated in *hpo* mutants [8]. With this background in mind, the presence of Notch was analysed in the ovaries of BgHpo knockdown females and, unlike in *D. melanogaster*, the activity of Notch was maintained. In the basal ovarian follicle of dsBgHpo-treated females, Notch is localized apically in the stalk and in the follicular epithelium at mitotic cycle but not during endocycle, and there is a gradient of NICD labelling, decreasing from the posterior pole of the oocyte towards the anterior pole. This kind of gradient has also been described in *D. melanogaster hpo* and *sav* mutants, as well as in those that overexpress *yki*, which also resulted in an accumulation of NICD in the posterior follicle cells [8], [11] despite their reduced Notch activity [7]–[9]. However, in contrast to *D. melanogaster hpo* mutants, in which reduced Notch activity and consequent egg chamber fusion has been described [8], [9], the depletion of Hippo mRNA in *B. germanica* results in an upregulation of Notch, as well as in an increase of the expression of Notch dependent genes. Given the phylogenetically basal position of *B. germanica*, these results suggest that the repression of Notch was an ancestral function of Hippo in insect ovaries.

Furthermore, the long-stalk phenotype observed in *B. germanica* Hippo-depleted females contrasts with the egg chamber fusion caused by the absence of stalks described in *hpo* mutants and in *yki*-overexpressing females of *D. melanogaster* [11]. Indeed, the long-stalk phenotype has been described in ovaries of *D. melanogaster* when active Notch and Delta are constitutively expressed, while in Notch mutants this particular phenotype is not observed [23], [24]. The present results indicate that the Notch pathway, and specifically Notch, is involved in the determination of stalk cell fate in panoistic ovaries and that the mechanism of Notch pathway activation differs from that manifested in meroistic ovaries. In *B. germanica*, Notch activates BgCut and BgEya, but BgHnt expression is not affected, suggesting that BgN does not act directly on BgHnt to regulate BgCut, as has been described in the ovaries of *D. melanogaster* [36], [37].

These evidences indicate that, in *B. germanica*, Hippo pathway is attenuating Notch in the follicular epithelium to control cell proliferation and in the stalk to maintain the correct number of stalk cells. This role of Notch, maintaining the mitotic cycle of somatic follicle cells, has been recently reported in the beetle *T. castaneum* [12], a basal holometabolan insect with meroistic telotrophic ovaries. Comparing the data obtained in *B. germanica* to those previously published from meroistic ovaries of *D. melanogaster* [34], [38] and *T. castaneum* [12], a clearer image is emerging about the functions of Notch and Hippo pathways during oogenesis in insects, and how have they changed during insect evolution. Taken together, the data suggest that in insect oogenesis Notch pathway has at least two functions: first, it is involved in maintaining the cells in an undifferentiated state, as in other developmental contexts. Upon Notch inactivation, follicular cells do not proliferate and they enter the endocycle prematurely giving rise to small ovarioles. This role of Notch seems to be ancestral as it has been found in the meroistic telotrophic ovaries of *T. castaneum* [12] and in the panoistic ovaries of *B. germanica*, but not in the meroistic polytrophic ovaries of *D. melanogaster* a highly modified insect species, where Notch has the opposite role [34], [39]. The second function of Notch is in the specification of stalk and polar cells, a function that has apparently been conserved across the different ovary types, as low levels of Notch activity are needed to maintain the correct structure of these cells [24], [40].

Regarding the Hippo pathway, in both *D. melanogaster* and *B. germanica*, Hippo signaling attenuates follicular cell proliferation by a differential regulation of Notch. As the panoistic ovaries are more ancestral, the results presented suggest that in insect ovaries, the role of hpo in *B. germanica* maybe the ancestral role.

## Supporting Information

Figure S1 **Follicular epithelium from 0-day-old adult females (A) and 5-day-old adult females (B).** The separate channels for F-actins (A′ and B′) and DNA staining (A″ and B″) from panels E and G of Figure 3 are showed.
(TIF)

Figure S2 **Bilayered follicular epithelium in dsBgHpo-treated female.** A: Optical section of a basal ovarian follicle from a 0-day-old adult female treated with dsMock. B: Optical section of a basal ovarian follicle from a 5-day-old adult female treated with dsBgHpo, showing the oocyte membrane (OM, arrow) folded, as it cannot be extended due to the bilayer of cells in the follicular epithelium (FE). The posterior end of the ovarian follicle is towards the left. Oo: Oocyte, Ov: Oviduct, P: Pedicel.
(TIF)

Figure S3 **BgWts controls ovarian follicle and stalk size regulating follicular cell proliferation.** A: Stalk from a 5-day-old adult dsMock-treated female showing the F-actin labelling. B: Follicular epithelium from a 5-day-old adult dsMock-treated female, showing large intercellular spaces. All the cells are large and binucleated. C: Ovariole from a 5-day-old adult dsBgWts-treated female. D: Long-stalk phenotype in a 5-day-old adult dsBgWts-treated female. E: Follicular epithelium from D, showing small mononucleated cells. F: Follicular epithelium from a 5-day-old adult dsBgWts-treated female showing mitosis, evidenced by the labelling with anti-PH3. The posterior end of the ovarian follicles is towards the bottom. Nucleus were stained with DAPI (blue in B and F, grey in C and E), and the F-actins (green) were stained with phalloidin-TRITC. Scale bar: 50 µm except in C that represents 100 µm. BOF: basal ovarian follicle, FE: Follicular epithelia, s: stalk, sBOF: subbasal ovarian follicle.
(TIF)

Table S1 **Primer sequence used for qRT-PCR and RNAi experiments.** The accession numbers of studied sequences are indicated. F: Primer forward. R: Primer reverse. In red are showed the housekeeping genes used in expression studies: BgActin-5c used in pattern expression and BgEIF4-a used in RNAi studies.
(DOCX)
