# Supplementary figures and images for: Unlike in Drosophila Meroistic Ovaries, Hippo Represses Notch in Blattella germanica Panoistic Ovaries, Triggering the Mitosis-Endocycle Switch in the Follicular Cells
Source: PLoS One. 2014 Nov 26;9(11):e113850. doi: 10.1371/journal.pone.0113850 (PMC4245235; doi:10.1371/journal.pone.0113850)

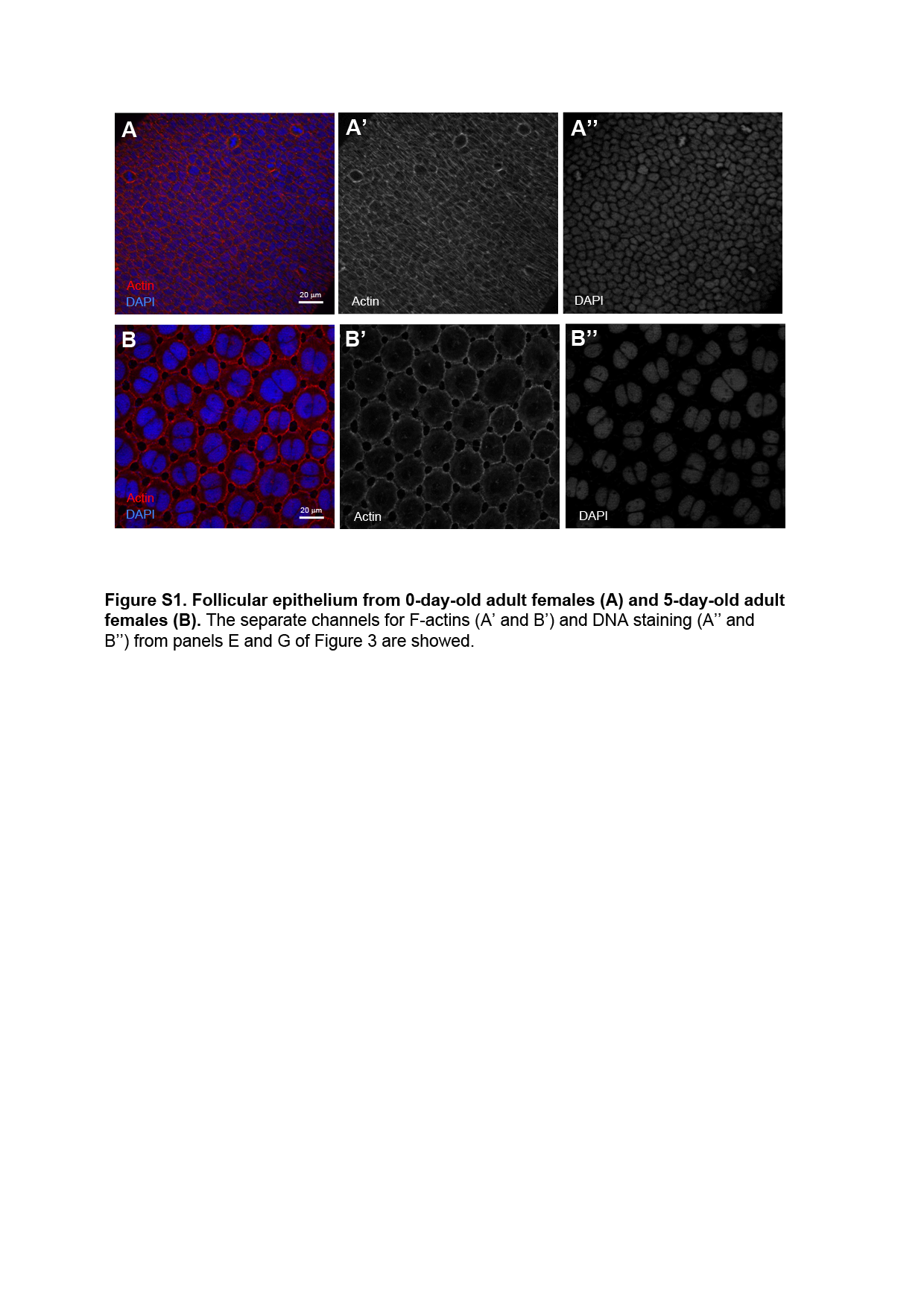

Supplement: Figure S1 — Follicular epithelium from 0-day-old adult females (A) and 5-day-old adult females (B). The separate channels for F-actins (A′ and B′) and DNA staining (A″ and B″) from panels E and G of Figure 3 are showed. (TIF) [file pone.0113850.s001.tif]

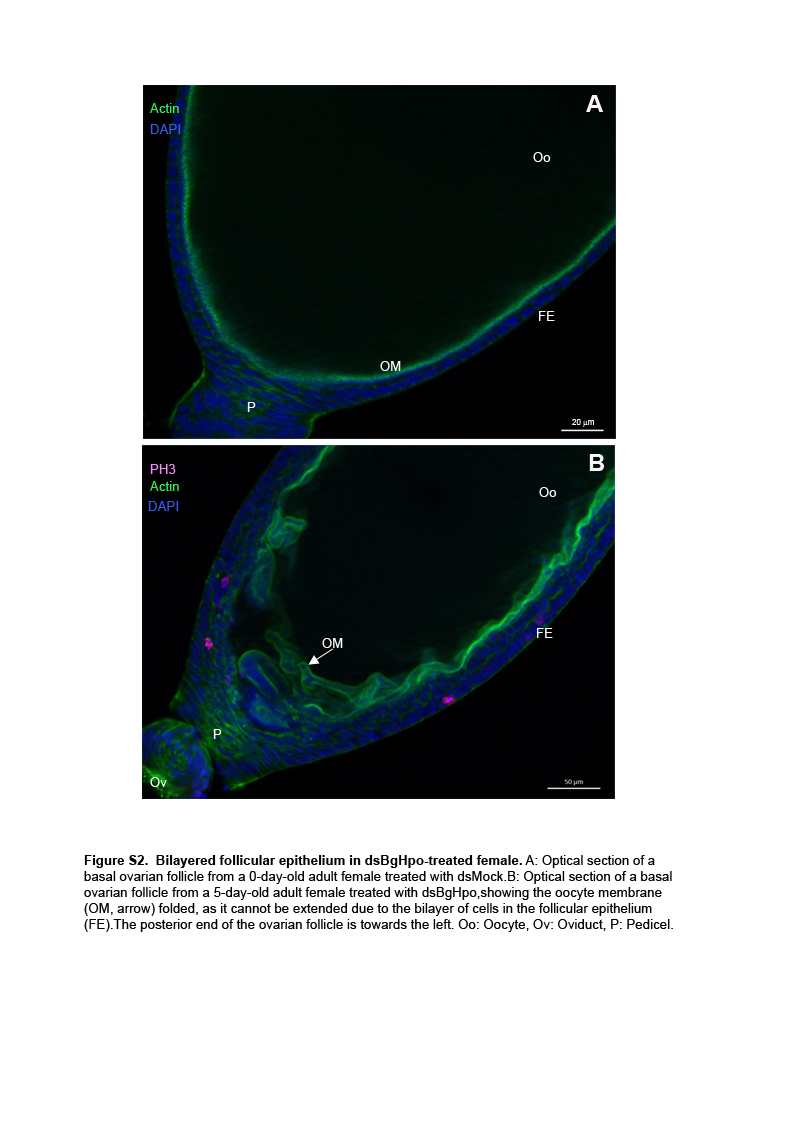

Supplement: Figure S2 — Bilayered follicular epithelium in dsBgHpo-treated female. A: Optical section of a basal ovarian follicle from a 0-day-old adult female treated with dsMock. B: Optical section of a basal ovarian follicle from a 5-day-old adult female treated with dsBgHpo, showing the oocyte membrane (OM, arrow) folded, as it cannot be extended due to the bilayer of cells in the follicular epithelium (FE). The posterior end of the ovarian follicle is towards the left. Oo: Oocyte, Ov: Oviduct, P: Pedicel. (TIF) [file pone.0113850.s002.tif]

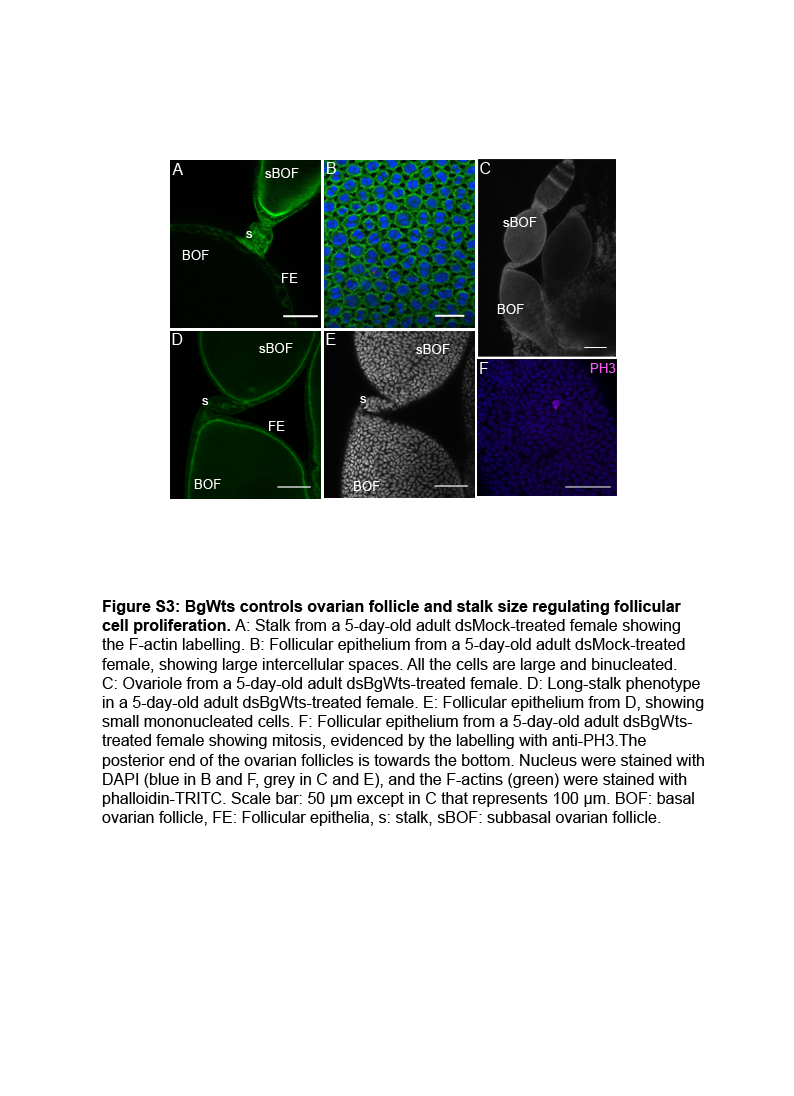

Supplement: Figure S3 — BgWts controls ovarian follicle and stalk size regulating follicular cell proliferation. A: Stalk from a 5-day-old adult dsMock-treated female showing the F-actin labelling. B: Follicular epithelium from a 5-day-old adult dsMock-treated female, showing large intercellular spaces. All the cells are large and binucleated. C: Ovariole from a 5-day-old adult dsBgWts-treated female. D: Long-stalk phenotype in a 5-day-old adult dsBgWts-treated female. E: Follicular epithelium from D, showing small mononucleated cells. F: Follicular epithelium from a 5-day-old adult dsBgWts-treated female showing mitosis, evidenced by the labelling with anti-PH3. The posterior end of the ovarian follicles is towards the bottom. Nucleus were stained with DAPI (blue in B and F, grey in C and E), and the F-actins (green) were stained with phalloidin-TRITC. Scale bar: 50 µm except in C that represents 100 µm. BOF: basal ovarian follicle, FE: Follicular epithelia, s: stalk, sBOF: subbasal ovarian follicle. (TIF) [file pone.0113850.s003.tif]
