# Supplementary material for: Unlike in Drosophila Meroistic Ovaries, Hippo Represses Notch in Blattella germanica Panoistic Ovaries, Triggering the Mitosis-Endocycle Switch in the Follicular Cells
Source: PLoS One. 2014 Nov 26;9(11):e113850. doi: 10.1371/journal.pone.0113850 (PMC4245235; doi:10.1371/journal.pone.0113850)
Supplement: Table S1 — Primer sequence used for qRT-PCR and RNAi experiments. The accession numbers of studied sequences are indicated. F: Primer forward. R: Primer reverse. In red are showed the housekeeping genes used in expression studies: BgActin-5c used in pattern expression and BgEIF4-a used in RNAi studies. (DOCX) [file pone.0113850.s004.docx]

**Table S1**: **Primer sequence used for qRT-PCR and RNAi experiments.** The accession numbers of studied sequences are indicated. F: Primer forward. R: Primer reverse. In red are showed the housekeeping genes used in expression studies: BgActin-5c used in pattern expression and EIF4-a used in RNAi studies.

|  | **Accession number** | **Primer name** |  | **Primer sequence** | **Amplicon length (bp)** |
| --- | --- | --- | --- | --- | --- |
| 1 | EMBL: HF969251 | BgHpo-RT | F  R | 5’-GACATTTGGAGCCTTGGCAT-3’  5’-AGGTTTCCCTTCAGCCATTTC-3’ | 51 |
| 2 | EMBL: HF969251 | BgHpo-RNAi-1 | F  R | 5’-GAACACTGACCTGTGGATTGT-3’  5’-AGGGTGAATATCTCCATATGG-3’ | 406 |
| 3 | EMBL: HF969251 | BgHpo-RNAi-2 | F  R | 5’-TACCGAGGAGGAGCCTACAA-3’  5’-TTCTCATGATGATGCCTTGC-3’ | 420 |
| 4 | HF969253 | BgYki-RT | F  R | 5’-TCCCTACCACACACACCAGA-3’  5’-GACCATCCAATGTTGCCATA-3’ | 103 |
| 5 | EMBL: HF969255 | BgN-RT | F  R | 5’-GCTAAGAGGCTGTTGGATGC-3’  5’-TGCCAGTGTTGTCCTGAGAG-3’ | 55 |
| 6 | EMBL: HF969255 | BgN-RNAi | F  R | 5’-CTCAGGACAACACTGGCAGA-3’  5’-AGGCTTCGTAACTGCCTTCA-3’ | 363 |
| 7 | EMBL: HF969264 | BgKibra-RT | F  R | 5’-AGCCACCGTCAGAAGTAACC-3’  5’-CATGAACTTTTTCCCGCACT-3’ | 96 |
| 8 | EMBL: HF969259 | BgMer-RT | F  R | 5’-CCGAAGCAGAACAGGAAATC-3’  5’-CTGCTTCCCTTGTCTTACGC-3’ | 93 |
| 9 | EMBL: HF969260 | BgExp-RT | F  R | 5’-CGCTACACAGGGAAAACCAT-3’  5’-ATGCCTCTCGGATGTACAGG-3’ | 66 |
| 10 | EMBL: HF969261 | BgMats-RT | F  R | 5’-TTGGGATCAGGAAATCTTCG-3’  5’-CCACAGTGTTCACAGCAACC-3’ | 82 |
| 11 | EMBL: HG007962 | BgWts-RT | F  R | 5’-CGTACAAGCAGCGAATGTTT-3’  5’-CAGGGCTCAGACGGATTTT-3’ | 69 |
| 12 | EMBL: HG007962 | BgWts-RNAi | F  R | 5’-AATGCGGAAAATGCTTTCAC-3’  5’-ACCCACTCATTGTCAGCCTC-3’ | 255 |
| 13 | EMBL: HF969262 | BgSav-RT | F  R | 5’-CAACCTTGTCAGCATTGCATTAA-3’  5’-ATCTAATTCCGGTAGCCTGAACAT-3’ | 51 |
| 14 | EMBL: HF969263 | BgSd-RT | F  R | 5’-GCCCACAGAGTGCTTTCTTC-3’  5’-CCCCTGCCTCATCTTGAATA-3’ | 72 |
| 15 | EMBL: CAA06379 | BgVg-RT | F  R | 5’-CTGGGCATTTGACAACACAACAT-3’  5’-TTGAAGAGCTGCTGGAGAGTTTG-3’ | 116 |
| 16 | EMBL: HF969265 | BgCut-RT | F  R | 5’-AAATATGTGCTCGGCCTGTC-3’  5’-TGCATCTTGCGGTAACTGTC-3’ | 107 |
| 17 | EMBL: HF969258 | BgHnt-RT | F  R | 5’-CTACGACATCGCAAGAAGCA-3’  5’-AAAGGGCAGTGGAGTTGTTG-3’ | 40 |
| 18 | EMBL: HF969257 | BgEya-RT | F  R | 5’-GGCTCTTAGGCACAAAACGA-3’  5’-GCAAGGGCTGGAACTAACTG-3’ | 166 |
| 19 | EMBL: HF969256 | BgDl-RT | F  R | 5’-CCACTACAAGTGTTCGCCAA-3’  5’-TACCTCTCGCATTCGTCACA-3’ | 180 |
| 20 | EMBL: HG515375 | BgSer-RT | F  R | 5’-TCCTCTTGGCAGTGCATTTG-3’  5’-CTTGATCACAGAGGATGCCG-3’ | 87 |
| 21 | EMBL: AJ862721 | BgActin-5c | F  R | 5’-AGCTTCCTGATGGTCAGGTGA-3’  5’-ACCATGTACCCTGGAATTGCCGACA-3’ | 213 |
| 22 | EMBL: HF969254 | BgEIF4a | F  R | 5’-ATGGTGACATGCCACAAAAA-3’  5’-GCAACACCTTTCCTTCCAAA-3’ | 208 |
